# Northern range expansion of the Asian tiger mosquito (*Aedes albopictus*): Analysis of mosquito data from Connecticut, USA

**DOI:** 10.1371/journal.pntd.0005623

**Published:** 2017-05-18

**Authors:** Philip M. Armstrong, Theodore G. Andreadis, John J. Shepard, Michael C. Thomas

**Affiliations:** Department of Environmental Sciences, Center for Vector Biology and Zoonotic Diseases, The Connecticut Agricultural Experiment Station, New Haven, Connecticut, United States of America; North Carolina State University, UNITED STATES

## Abstract

**Background:**

The Asian tiger mosquito (*Aedes albopictus*) is an invasive species and important arbovirus vector that was introduced into the U.S. in the 1980's where it continues to expand its range. Winter temperature is an important constraint to its northward expansion, with potential range limits located between the 0° and -5°C mean cold month isotherm. Connecticut is located within this climatic zone and therefore, *Ae*. *albopictus* was monitored statewide to assess its northern range expansion and to delineate where populations can stably persist.

**Methodology/Principal findings:**

*Ae*. *albopictus* females were monitored at fixed trapping sites throughout Connecticut from June-October over a 20-year period, 1997–2016. In addition, *Ae*. *albopictus* larvae and pupae were collected from tire habitats and tires were retrieved from the field in the spring and flooded to evaluate overwintering success of hatching larvae. *Ae*. *albopictus* was first detected during statewide surveillance when a single adult female was collected in 2006. This species was not collected again until 2010 and was subsequently detected each successive year with increasing abundance and distribution except following the unusually cold winters of 2014 and 2015. *Ae*. *albopictus* mosquitoes were most abundant in urban and suburban locations along the southwestern shoreline of Connecticut; however, single specimens were occasionally detected in central parts of the state. Field-collected females were also screened for arbovirus infection yielding two isolations of Cache Valley virus and one isolation of West Nile virus, highlighting the threat posed by this mosquito. *Ae*. *albopictus* overwintered in Connecticut under mild winter conditions as shown by recovery of hatched larvae from field collected tires in spring and by early season detection of larvae and pupae.

**Conclusions/Significance:**

This study documents the establishment and expansion of *Ae*. *albopictus* at the northern boundary of its range in the northeastern U.S. and provides a baseline for monitoring the future spread of this species anticipated under climate change.

## Author summary

The Asian tiger mosquito (*Aedes albopictus*) is a highly invasive species and an important disease vector that is undergoing rapid range expansion in many countries including the U.S. Winter temperature is an important limit to its northward expansion with Connecticut situated near the northern boundary of its potential range. In this study, we sampled mosquitoes at fixed trapping sites located statewide to track the establishment and range expansion of *Ae*. *albopictus* in this region. In addition, mosquito larvae were monitored in tire habitats to evaluate overwintering success of local populations. From this effort, we describe the initial detection of *Ae*. *albopictus* in 2006, its annual reemergence and population expansion in southwestern Connecticut from 2010–2016, and its local overwintering under mild winter conditions. Together, this study documents population changes in *Ae*. *albopictus* at the northern boundary of its range and provides a baseline for monitoring future range expansion anticipated under climate change.

## Introduction

The Asian tiger mosquito, *Aedes albopictus* (Skuse), is an invasive species that has spread from East Asia to the Americas, Africa, Europe, and the Middle East primarily by the global tire trade [1, 2]. This mosquito inhabits urbanized environments [3], develops in artificial, water-holding containers [4], and feeds readily on humans, making it a major pest species throughout its range [5–7]. *Ae*. *albopictus* is also an important vector of dengue virus (DENV), chikungunya virus (CHIKV), and Zika virus (ZIKV) in many parts of the world. This includes its primary role in outbreaks of DENV in China [8], CHIKV in the Indian Ocean Islands [9], ZIKV in central Africa [10], and autochthonous transmission of DENV and CHIKV in southern Europe [11–13]. In addition, *Ae*. *albopictus* is competent to transmit 23 other arboviruses [14]. This includes eastern equine encephalitis virus, West Nile virus (WNV), and La Crosse virus that have also been isolated from this species during field investigations in the U.S. [3, 15, 16].

In the continental U.S., the first established population of *Ae*. *albopictus* was documented in Houston, Texas in 1985 [17], followed by its rapid expansion throughout southeastern and parts of northeastern and northcentral U.S. [2, 18]. *Ae*. *albopictus* has been occasionally collected in locations as far north as Minnesota, New Hampshire, and Ontario, Canada, but these records most likely represent seasonal introductions rather than permanent resident populations [19, 20]. The northern distribution limit for overwintering populations was conservatively estimated to be at the 0°C cold-month isotherm based on its distribution in Asia [21]. The southern coast of Connecticut is at this thermal limit of *Ae*. *albopictus* and possesses suitable habitat for colonization by this species. In 2003, *Ae*. *albopictus* (a single female) was first collected during field studies in southern Connecticut but was not further detected that same year during statewide mosquito surveillance [22]. An infestation of this species was then discovered at a tire recycling plant in northeastern Connecticut in 2006, yet the population failed to overwinter and establish at the site [23]. Clearly, continued monitoring for this species is warranted in this region, particularly in urban and suburban locations and under milder winter conditions that are anticipated to increase in frequency due to climate change.

In this study, we document the northern range expansion of *Ae*. *albopictus* by continuous monitoring of mosquito populations over a 20 year period in Connecticut. Mosquitoes were collected at 91 fixed trapping locations from June-October, identified to the species level, and tested for arbovirus infection as a part of the statewide mosquito arbovirus surveillance program. Mosquito larvae and pupae were also monitored in a used tire habitat in Stratford, (Fairfield County) Connecticut at a known locality for *Ae*. *albopictus* reproduction. Tires were also retrieved from the field in the spring and flooded to evaluate overwintering success of *Ae*. *albopictus*. From this effort, we describe the initial detection of *Ae*. *albopictus* in 2006, its annual reemergence and population expansion in the state from 2010–2016, and its local overwintering under mild winter conditions.

## Methods

### Adult sampling

**Fig 1:**
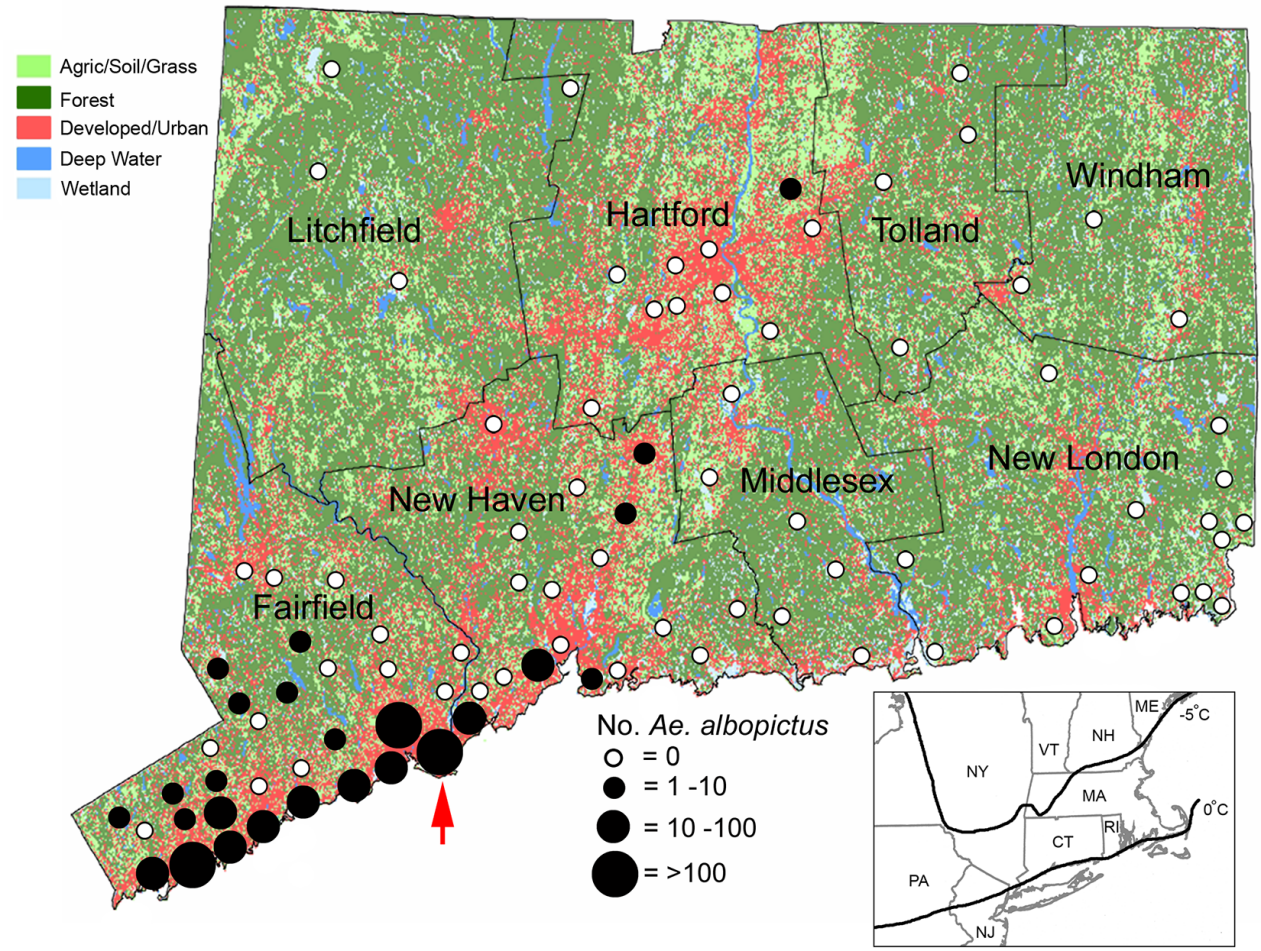
County map of Connecticut showing geographic distribution of mosquito trapping sites and land use characteristics. Black circles indicate locations where *Ae*. *albopictus* were collected from 2006–2016 and are scaled according to numbers collected at each site. Red arrow indicates location of the Stratford site. Inset map of northeastern U.S. shows approximate location of the 0°C and -5°C cold month isotherm.

**Fig 2:**
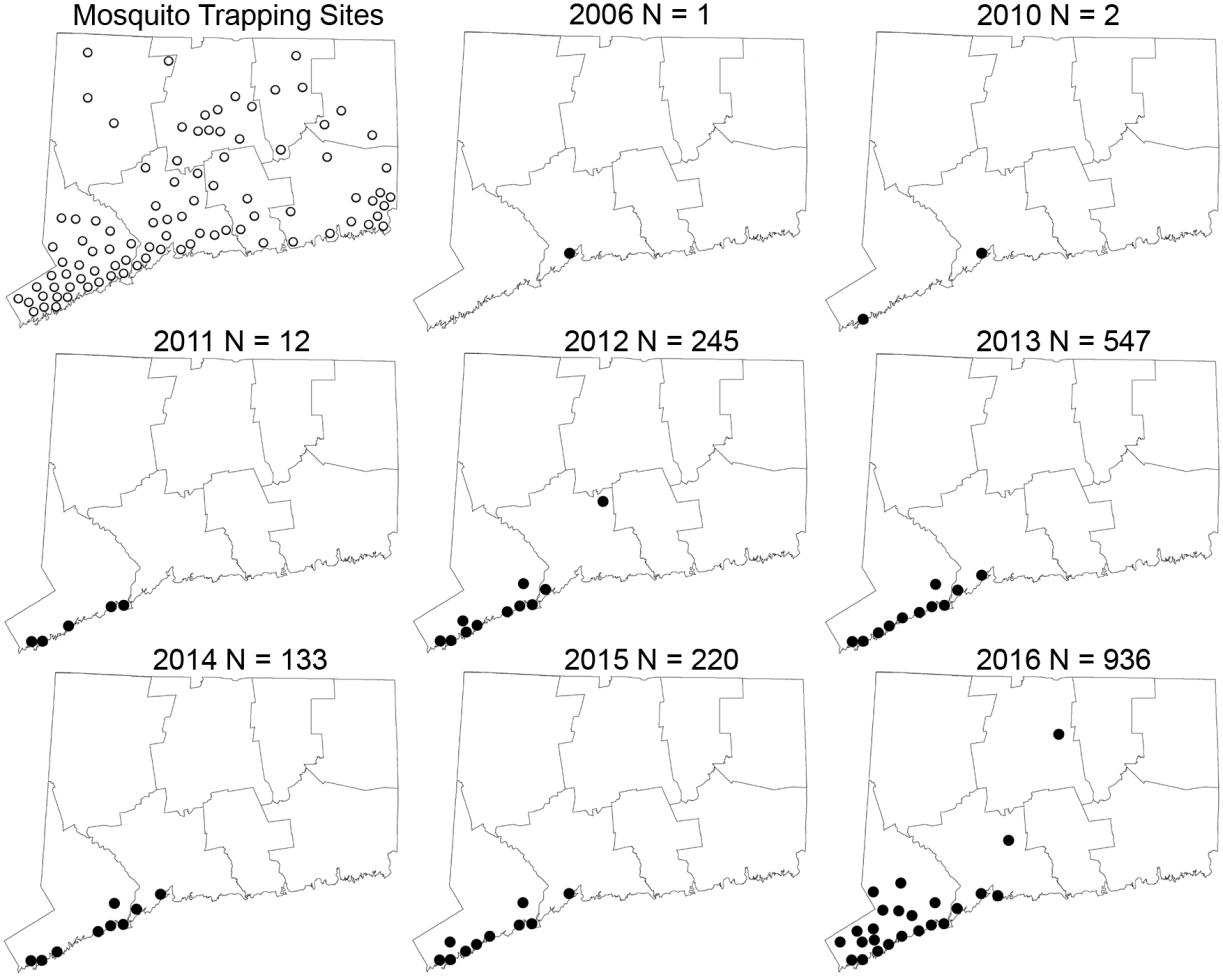
Connecticut map showing geographic locations where *Ae*. *albopictus* were collected by year. Open circles indicate location of all mosquito trapping sites and black circles depict trapping sites positive for *Ae*. *albopictus*. Number of *Ae*. *albopictus* collected is indicated for each year.

Mosquitoes were collected at 91 trapping locations statewide (Figs 1 and 2) as a part of the Connecticut Mosquito and Arbovirus Surveillance Program from June through October [24]. Thirty-six of the sites have been monitored since 1997 with the balance of sites in continuous operation since 2000. About half of the sites are located in surburban and urban locations, primarily in the southwestern and central parts of the state, which are focal areas for WNV. Specific trapping locales include neighborhood parks, school grounds, sewage treatment plants, municipal dumping stations, industrial parks, and fragmented woodlots. The remaining sites are located in more rural settings that are associated with eastern equine encephalitis virus transmission and include permanent fresh-water swamps and bogs, coastal salt marshes, and mixed woodlands.

Each trapping site was sampled on average weekly and at least once every 10 days by setting a CO_2_-baited CDC light trap and a gravid trap (John W. Hock Co., Gainesville, FL) baited with a hay/yeast/lactalbumin infusion [25]. In addition, BG Sentinel (BGS) traps baited with the BG–Lure but without CO_2_ (Biogents AG, Regensburg, Germany) were added after the detection of *Ae*. *albopictus* at a site. Traps were operated overnight and adult mosquitoes were transported back to the laboratory alive in an ice chest. Mosquitoes were immobilized with dry ice, sorted, and identified to species on chill tables with the aid of a stereo microscope and taxonomic keys [22]. Female mosquitoes were combined into pools of 50 or fewer individuals by location, trap type, species, and collection date in 2 mL microcentrifuge tubes containing a copper BB, and stored at -80°C until virus testing.

### Virus detection

Mosquito pools were prepared for virus isolation by adding 1 mL of PBS-G (phosphate buffered saline, 30% heat-inactivated rabbit serum, 0.5% gelatin, and antibiotic/antimycotic) to each tube. Mosquito pools were homogenized using the MM300 Mixer Mill (Retsch Laboratory, Hann, Germany) set for 4 min at 25 cycles/sec. Mosquito homogenates were centrifuged for 6 minutes at 7,000 rpm at 4°C and 100 μL of the supernatant was inoculated into Vero cell cultures- clone E6 (provided by Shirley Tirrell, Yale University) growing in 25 cm^2^ flasks. Vero cells were maintained at 37°C and 5% CO_2_ and examined daily for cytopathic effect from days 3–7 post-inoculation. RNA was extracted from infected cell supernatants and the corresponding mosquito pool using the viral RNA kit (Qiagen, Valencia, CA). Virus isolates were identified as WNV or Cache Valley virus (CVV) using PCR-based assays as previously described [26, 27].

### Larval surveys

From 2013–2016, larvae and pupae were sampled from a location in Stratford, Connecticut. This site is a small woodlot, located in a mixed residential and commercial neighborhood with an abandoned pile of about 30 waste tires concealed in the woods. The majority of the tires at the pile were not available for oviposition and larval development because they were buried or completely filled with organic material. Seven tires were flagged, positioned upright, and sampled weekly from May-October. Water samples were removed from tires with a small plastic container and fresh water was added back to maintain water levels in the tires. Mosquito larvae were identified using taxonomic keys under a compound microscope and pupae were allowed to emerge as adults prior to their identification.

To evaluate mosquito overwintering from 2013–2016, four tires were retrieved from the Stratford site in late April, and residual water and sediments were removed and inspected for larvae. The tires were then placed in a greenhouse, exposed to natural light and photoperiod, oriented in the same position as in the field, and flooded with water. Tires were inspected daily and larvae were identified as described above. The tires were then emptied and re-flooded a second time after a 2–3 week period when larvae were no longer observed, to evaluate if there was a delayed hatch.

### Data analysis

To assess the most effective method for collecting *Ae*. *albopictus*, we compared numbers of females collected in CDC-light, BGS, and gravid traps. Trap evaluations were performed only when all three trap types were in operation from 2010–2016 (n = 474 trap-nights). The mean number of *Ae*. *albopictus* collected per trap night was calculated for each trap type and compared by Krustal-Wallis, one-way analysis of variance (ANOVA) of ranks (Prism 7.0, GraphPad software). Dunn's multiple comparison post-tests were then applied to identify which pairs of trap types significantly differed from each other.

**Fig 3:**
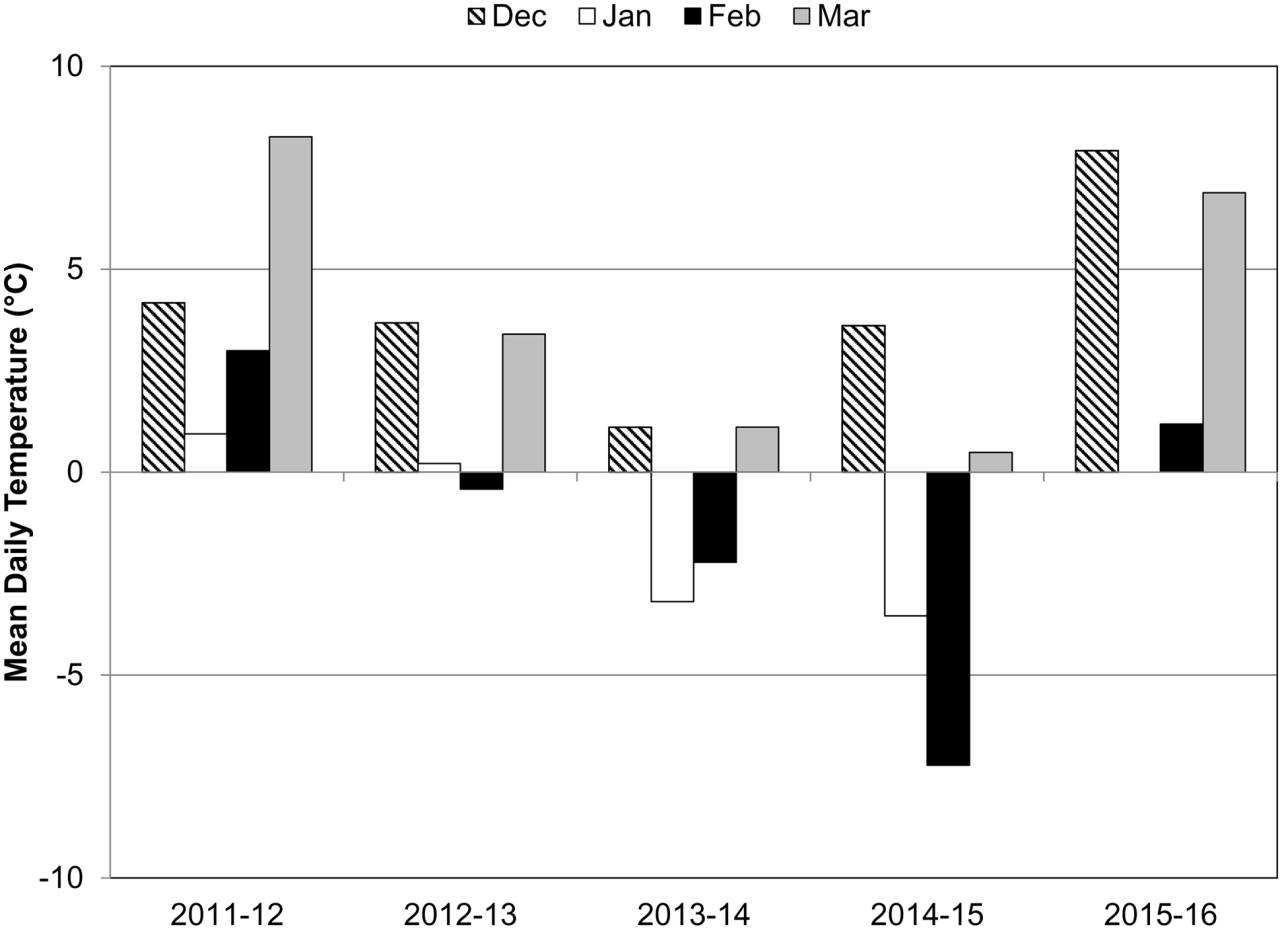
Mean monthly temperatures recorded at weather stations in Bridgeport, New Haven, Stamford, and Stratford, Connecticut.

Daily weather summaries were obtained from the National Oceanic and Atmospheric Administration National Centers for Environmental Information (https://www.ncdc.noaa.gov/cdo-web/datatools/selectlocation) for four locations in coastal Fairfield and New Haven counties: Bridgeport, Success Hill (41.200°N, 73.157°W), Stamford, 5N (41.125°N, 73.548°W), Stratford, Sikorsky Airport (41.158°N, 73.129°W), and New Haven, Tweed Airport (41.264°N, 72.887°W). Mean monthly temperatures were calculated for the winter months (Fig 3), and the lowest temperature recorded at each site per month was noted.

## Results

### Mosquito collection and virus detection

Figs 1 and 2 depict the spatial-temporal distribution and abundance of female *Ae*. *albopictus* collected during statewide mosquito trapping efforts in Connecticut. The first female *Ae*. *albopictus* was collected from a gravid trap located in New Haven County during 2006, with no additional collections until 2010 (N = 2) and 2011 (N = 12). The number of *Ae*. *albopictus* increased substantially during 2012 (N = 245, 11 locations) and 2013 (N = 547, 11 locations). During these years, positive sites were located in southwestern coastal Connecticut with the exception of one adult female collected in the central part of the state. *Ae*. *albopictus* collections decreased during 2014 (N = 133, 9 locations) and 2015 (N = 220, 10 locations) following winters with mean monthly temperatures well below the 0°C threshold and then increased sharply during 2016 (N = 936, 24 locations) following a more moderate winter (Fig 3). *Ae*. *albopictus* were detected primarily in densely-populated communities along the southwestern portion of the state; however, single specimens were also collected from a few sites in central Connecticut.

To evaluate the effectiveness of commonly implemented mosquito traps and methodologies, we compared *Ae*.*albopictus* collections for each trap configuration used in this study. The mean number of *Ae*. *albopictus* collected per trap night differed among the BGS trap with BG Lure (mean = 1.6/trap night), CDC light trap with CO_2_ (mean = 1.1/trap night) and gravid trap with hay infusion (mean = 0.4/trap night) (Krustal-Wallis ANOVA p<0.0001). Pairwise comparisons revealed significant differences in trapping efficiency between the BGS trap and gravid trap (Dunn's post-test p<0.0001) and the CDC-light trap and gravid trap (p<0.01), but not between the BGS and CDC-light traps (p = 0.7).

Female *Ae*. *albopictus* were processed and screened for arbovirus infection by Vero cell culture assay. There were two isolations of CVV from *Ae*. *albopictus* collected in Stratford in 2014 and one isolation of WNV from Bridgeport in 2016. WNV infection was then reconfirmed by directly testing the positive mosquito pool by real-time RT-PCR (Ct = 22.9).

### Seasonal abundance

**Fig 4:**
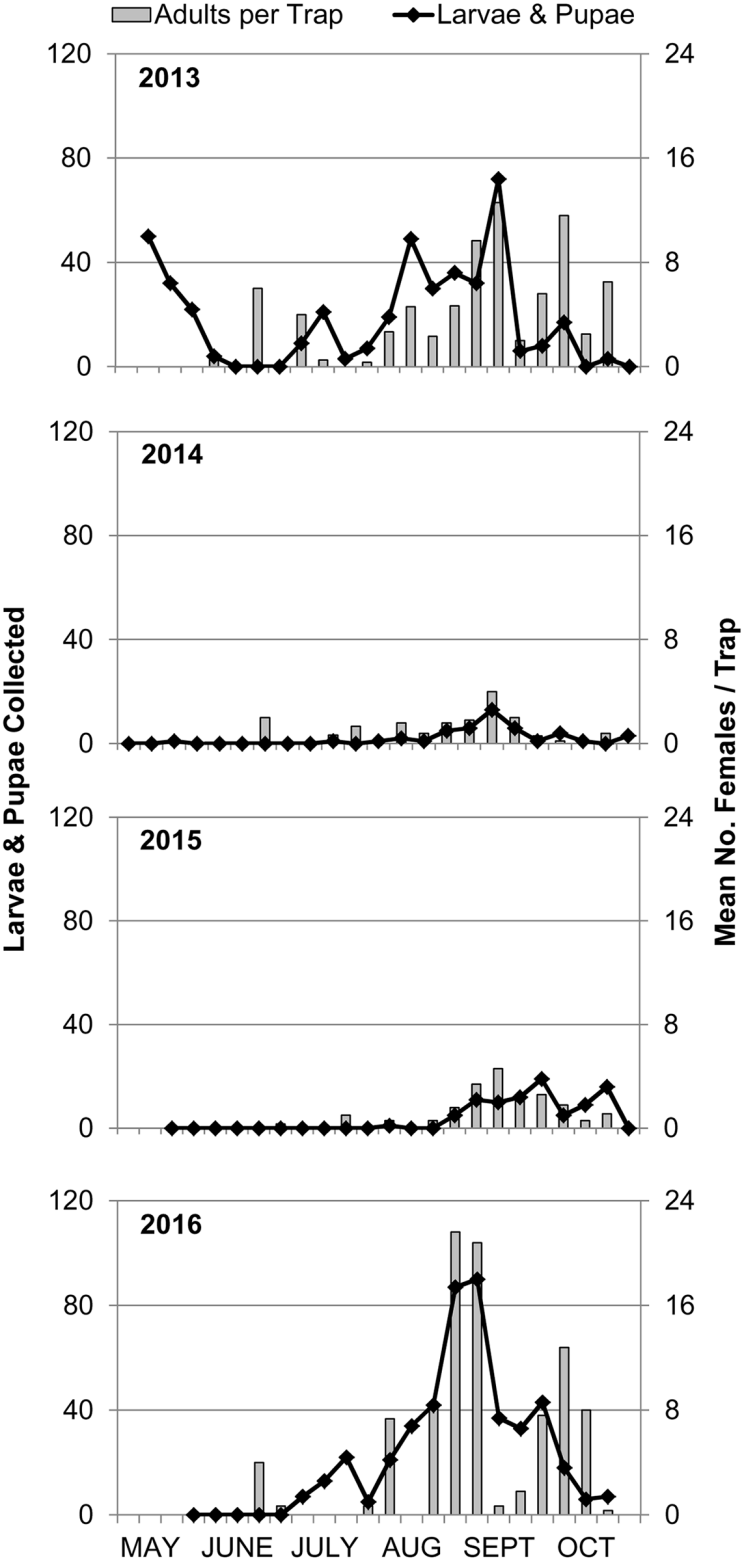
Weekly collection of juvenile (larvae and pupae) and adult (females) *Ae*. *albopictus* from Stratford, CT.

*Ae*. *albopictus* larvae and pupae were sampled weekly from tire habitats in Stratford, CT and compared to adult trap collections from the same site (Fig 4). Larvae and pupae were collected much earlier in the season during 2013 following a mild winter than in subsequent years (Fig 3). During 2013, the numbers of juvenile *Ae*. *albopictus* fluctuated throughout the season with peak abundances occurring during early May, August, and September. Adult collections lagged behind the larval- and pupal-cohorts with peaks occurring in early and late September. During 2014 and 2015, *Ae*. *albopictus* immatures and adults closely tracked each other, appearing later in the season and in fewer numbers than during 2013. *Ae*. *albopictus* populations rebounded during 2016; however, larvae appeared later in the season starting in July and peaked in late August to early September in parallel with adult collections.

### Evaluation of mosquito overwintering

To determine whether *Ae*. *albopictus* may survive winters in Connecticut, we retrieved four tires from the Stratford site in April and flooded them twice with water to recover hatched larvae. A total of 51 *Ae*. *albopictus* larvae were recovered during 2013, but none were collected from tires retrieved from the site in late April during 2014, 2015, or 2016. Other mosquito species hatching from the tires included *Aedes japonicus* (Theobald) (2013 N = 39 and 2014 N = 5) and *Aedes triseriatus* (Coquillett) (2013 N = 60 and 2014 N = 22); these species were not enumerated during 2015 and 2016.

## Discussion

This study documents the establishment and expansion of *Ae*. *albopictus* at the northern boundary of its range in southern New England. This species was first detected during statewide mosquito surveillance in 2006 and then reemerged every summer starting in 2010. *Ae*. *albopictus* mosquitoes were most abundant in urban and suburban locations along the shoreline of southwestern Connecticut; however, single specimens were occasionally detected in central parts of the state. Mosquito abundance and the number of positive traps increased every year starting in 2010 except following the cold winters of 2013–2014 and 2014–2015. These changes in abundance and distribution are not an artifact of sampling effort because mosquito populations were consistently sampled at fixed trapping sites over a 20 year period. We conclude that *Ae*. *albopictus* is expanding northward in the northeastern U.S. and this trend is anticipated to accelerate under conditions of climate change as previously noted [28, 29].

We also showed that *Ae*. *albopictus* was able to overwinter in Connecticut during 2012–2013. Local overwintering was demonstrated by recovery of larvae hatching in late April from tires that were left outside during winter and by early seasonal detection of *Ae*. *albopictus* larvae and pupae. This finding, however, could not be reproduced during the following two winters that were exceptionally cold (January and February mean temperatures -2.2 to -7.2°C) or following a more mild winter during 2015–2016 (Fig 3). The performance of overwintering populations is affected by the number diapause-conditioned eggs laid during the previous fall, and overwintering temperatures and conditions impacting egg mortality. The lack of evidence for overwintering success observed in the spring of 2016 following a warmer winter may be explained, in part, by a lower abundance of females in the previous fall, leading to a lower number of diapaused eggs deposited for overwintering. It is also noteworthy that temperatures dropped to an absolute low of -21°C during a cold snap in February 2016 despite warmer mean temperatures for the entire month as shown in Fig 3. This is well below the 50% survival threshold estimated for diapausing *Ae*. *albopictus* eggs which ranges from -5 to -13°C for a 24 hour exposure period and approaches the -26°C supercooling point that is 100% lethal under brief exposures [30, 31].

The primary mechanism driving the annual reemergence of *Ae*. *albopictus* in Connecticut is unknown, but may be due to local overwintering of mosquito eggs, annual reintroduction of mosquitoes from southern locations, or some combination of both of these processes. Environmental conditions appeared to be lethal for diapausing eggs during three of the four years of this study based on recovery of *Ae*. *albopictus* from overwintering tires during the spring. Nevertheless, we cannot preclude the possibility that this species may survive at low levels in microhabitats that were not represented in our study. *Ae*. *albopictus* eggs were shown to survive winter temperatures as low as -19°C in northern Indiana, presumably due to the insulating effects of snow cover [32]. Snow drifts, sheltered structures, and urban heat transfers may enhance overwintering survival of this species at the northern distribution limit. Further studies on the field survival of diapause eggs under different microhabitat conditions and temperature regimes are needed to better understand the conditions affecting its overwintering success.

*Ae*. *albopictus* is readily dispersed over long distances by the transport of dormant eggs and emergent larvae and pupae in tires and other water-holding containers [33, 34]. This may be an important mechanism for reestablishing populations after severe winter conditions. Potential reintroduction would most likely be human mediated as *Ae*. *albopictus* is a short distance disperser where most females remain within 300 m of the larval habitat from which they emerge [35]. The observation of late season emergence of *Ae*. *albopictus* in our study sites may reflect either reintroduction of mosquitoes from more southerly locations or perhaps low levels of mosquito overwintering and hatching in the spring that could not be readily detected until later in the summer when the populations rebounded. We cannot distinguish from these possibilities based on our current data but show direct evidence for mosquito overwintering at least under mild winter conditions.

A number of studies have estimated the geographic distribution of *Ae*. *albopictus* by correlating current thermal conditions with mosquito distribution records and using this data to develop spatial models to predict its potential range. Our findings are in close agreement with predicted northern range limits along the southern margin of Connecticut, Rhode Island, and Massachusetts based on mean annual and winter temperatures [21, 28, 36]. Other studies show the distribution limit extending further north well into New England, including most of Connecticut [37, 38], yet we did not find stable populations in the interior part of the state. Mean winter temperature was identified as the most significant environmental factor predicting its current range within northeastern U.S. [28], with thermal limits estimated between the 0°C and -5°C cold month isotherm [21, 36]. The observed distribution of *Ae*. *albopictus* populations in Connecticut aligns more closely to the 0°C isotherm; however, we anticipate future range expansion as founding populations continue to build, along with milder winters and hotter summers that are projected to increase in frequency under climate change [39].

Our current trapping methods for mosquito monitoring rely mainly on the deployment of CO_2_-baited CDC light and gravid traps. CDC light traps are effective for collecting a diversity of crepuscular and nocturnal feeding Anopheline and Culicine species, whereas gravid traps are more effective for trapping gravid *Culex* species. Neither trap type is particularly effective for collecting diurnal feeding and container-inhabiting *Aedes* species so we supplemented our collections by deploying BGS traps that have shown promise for the collection of *Ae*. *albopictus*. In this study, the BGS trap did not significantly improve capture of *Ae*. *albopictus* over the CDC light trap but both of these traps were significantly better than the gravid trap. These findings contrast with trap evaluations that were performed in locations with more established *Ae*. *albopictus* populations [40, 41]. In these studies, BGS traps outperformed CDC light traps that may be explained by their combined use of CO_2_ and BG-lures and/or the overall density of *Ae*. *albopictus* at the trapping site.

In this study, we isolated WNV and CVV from *Ae*. *albopictus* collected in Connecticut, raising concerns about its role as a potential arbovirus vector. Both of these arboviruses have been detected in field-collected *Ae*. *albopictus* from other states, including Pennsylvania and New Jersey [3, 42, 43]. *Ae*. *albopictus* has also been implicated as a potential bridge vector of WNV based on its vector competence [44] and feeding behavior that occasionally includes blood meals from virus-competent birds [45, 46]. However, in another study, WNV infection was not detected in more than 30,000 *Ae*. *albopictus* collected in New Jersey despite concurrent WNV amplification in the region, suggesting a limited role for this species as a vector [43]. It is striking that we isolated both CVV and WNV from a limited sample of *Ae*. *albopictus*, highlighting its potential threat, but further sampling and testing are required to assess its overall contribution. In addition, exotic arboviruses such as CHIKV, DENV, and ZIKV are frequently introduced into the U.S. by infected travelers returning from endemic countries necessitating research on the vector competence and capacity of local *Ae*. *albopictus* populations for these pathogens. This is an important priority given the central role that this species had in the autochthonous transmission of DENV and CHIKV in unlikely places such as Hawaii, France, Italy, and Japan [11, 12, 47, 48].
